# Supplementary material for: Navigating the Semiochemical Landscape: Attraction of Subcortical Beetle Communities to Bark Beetle Pheromones, Fungal and Host Tree Volatiles
Source: Insects. 2025 Jan 9;16(1):57. doi: 10.3390/insects16010057 (PMC11766014; doi:10.3390/insects16010057)

Supplementary materials

Figure S1. Percent of total trap catches of subcortical beetles in each treatment within Experiment 1: Fungal volatiles (FVOCs) without the addition of mountain pine beetle (MPB) pheromone. Traps were baited with seven different FVOC treatments, including 2-methyl-1-butanol (2M1B), 2-methyl-2-butanol (2M2B), 3-methyl-1-butanol (3M1B), acetoin (Ace), a synthetic blend of FVOCs (Blend), and isobutanol (Iso). The control treatment represents traps baited with mineral oil without the addition of fungal volatiles. Scolytinae were not included due to negligible catches.

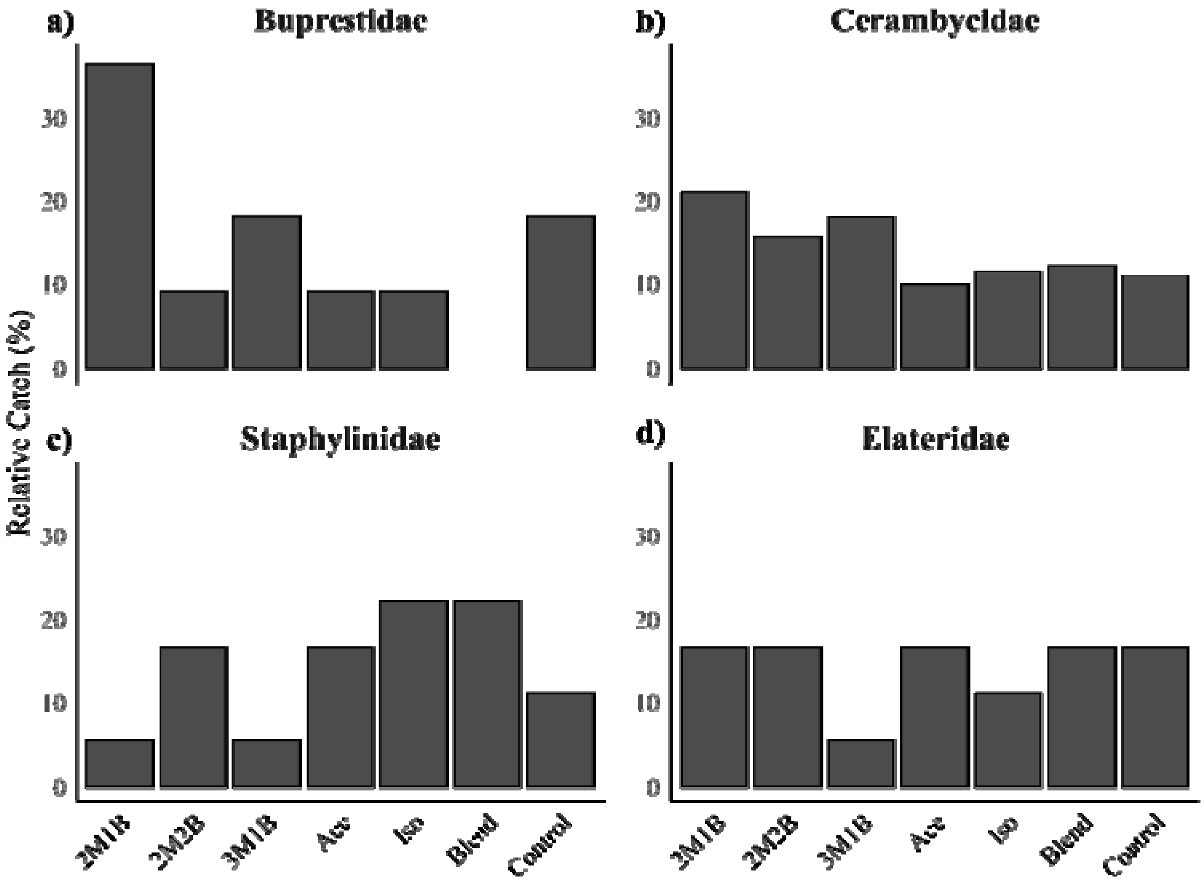

Figure S2. Percent of total trap catches of subcortical beetles in each treatment within Experiment 2: fungal volatiles (FVOCs) with the addition of mountain pine beetle (MPB) pheromone lures. Traps were baited with seven different FVOC treatments, including 2-methyl-1-butanol (2M1B), 2-methyl-2-butanol (2M2B), 3-methyl-1-butanol (3M1B), acetoin (Ace), a synthetic blend of FVOCs (Blend), and isobutanol (Iso). The control treatment represents traps baited with MPB pheromone lures without the addition of FVOCs.

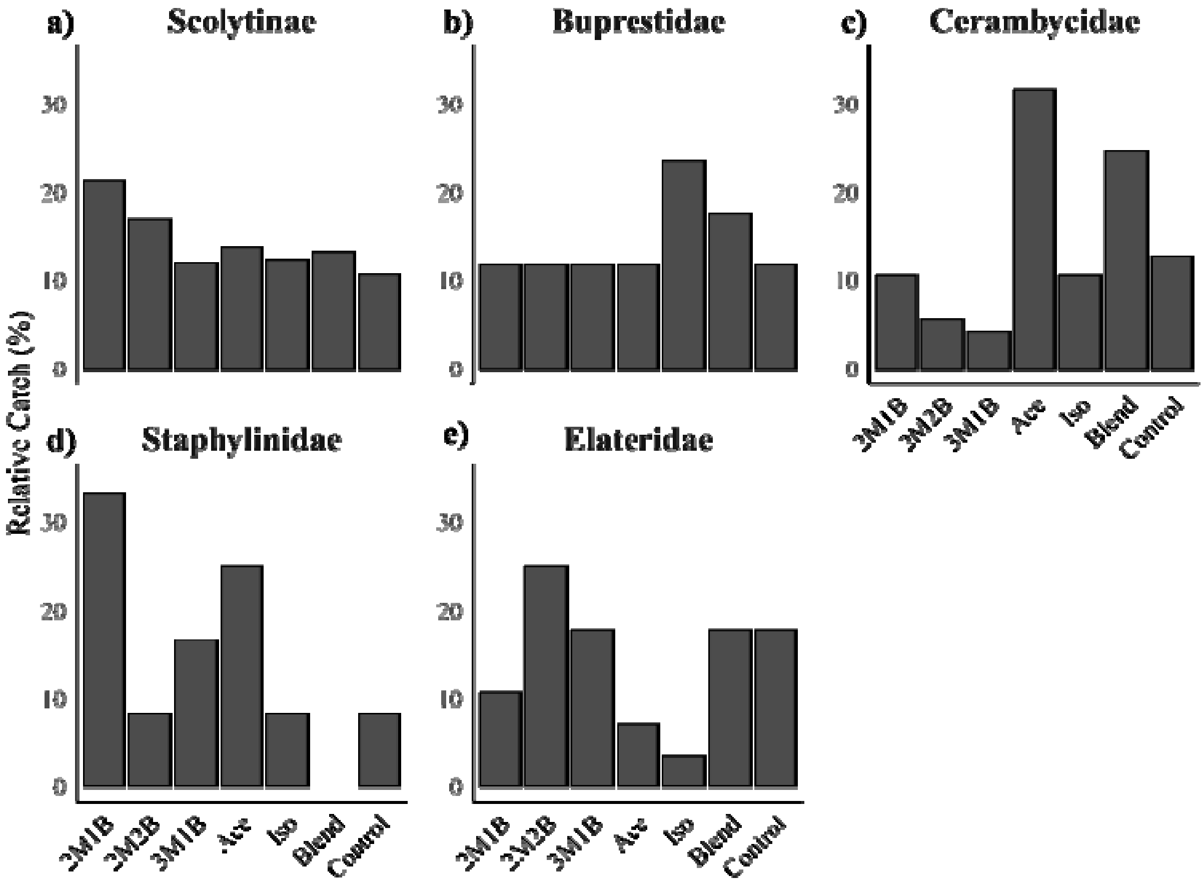

Figure S3. Percent of total trap catches of subcortical beetles in each treatment within Experiment 3: Host stress volatiles (SVCs) without the addition of mountain pine beetle (MPB) pheromone lures. The SVC treatments were chemical blends based on the volatile profiles associated with *Ophiostoma montium* (OM), *Leptographium longiclavatum* (LL), *Grosmannia clavigera* (GC), *Atropellis piniphila* (AP), *Endocronartium harknessii* (EH) and the profile of a healthy lodgepole pine (Healthy). The control treatment represents traps baited with mineral oil without the addition of host stress volatiles.

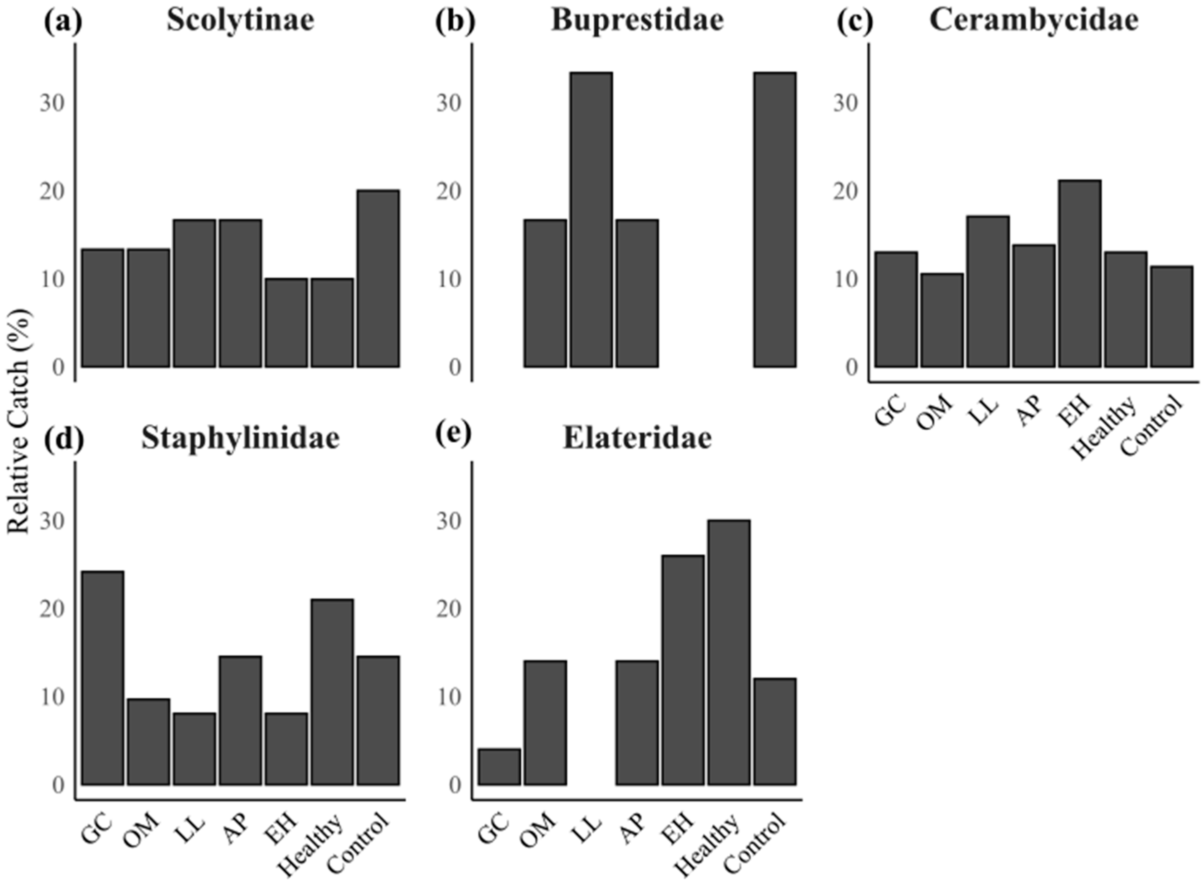

Supplement: Supplementary file 1 [file insects-16-00057-s001.zip › supplementary_materials/Supplementary materials.pdf]
