# Supplementary figures and images for: Navigating the Semiochemical Landscape: Attraction of Subcortical Beetle Communities to Bark Beetle Pheromones, Fungal and Host Tree Volatiles
Source: Insects. 2025 Jan 9;16(1):57. doi: 10.3390/insects16010057 (PMC11766014; doi:10.3390/insects16010057)

**(a) Buprestidae**

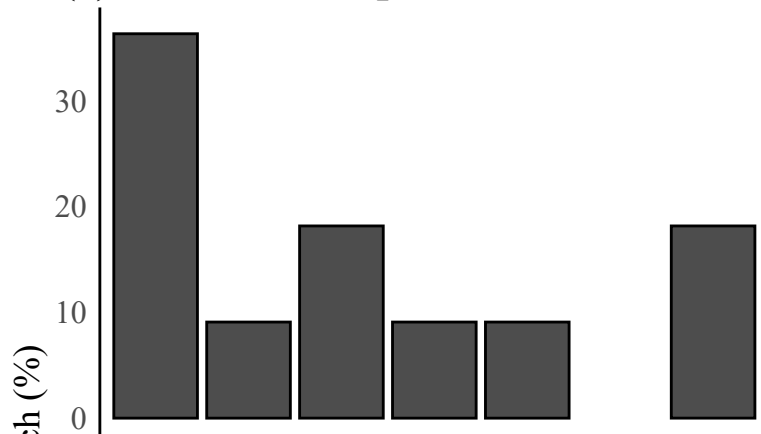

**(b) Cerambycidae**

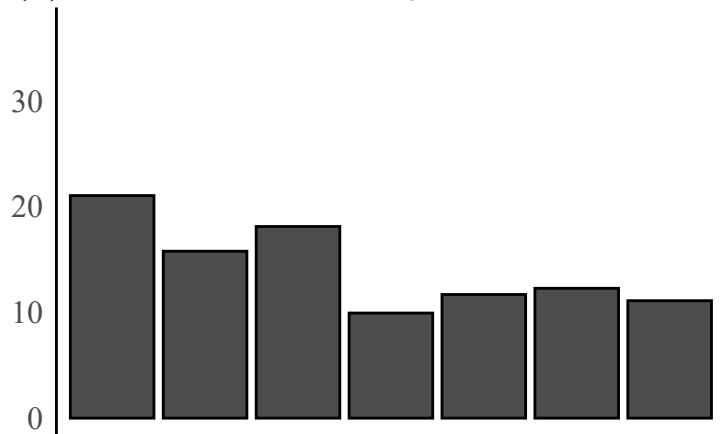

**(c) Staphylinidae**

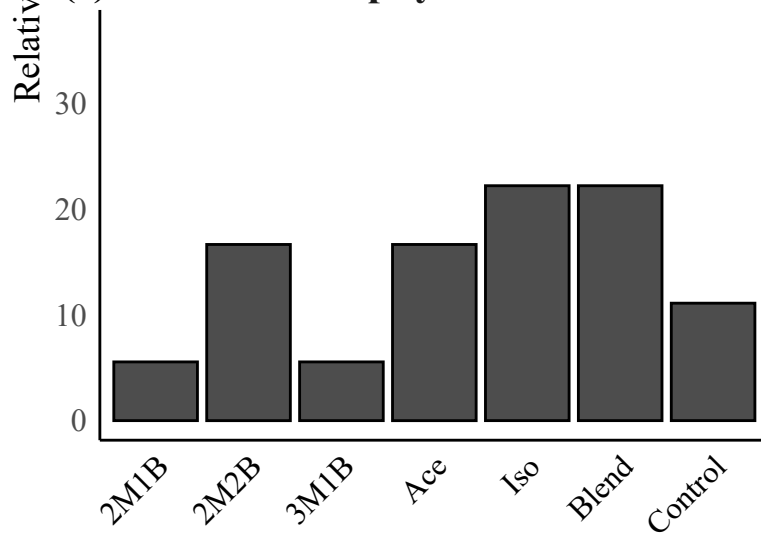

**(d) Elateridae**

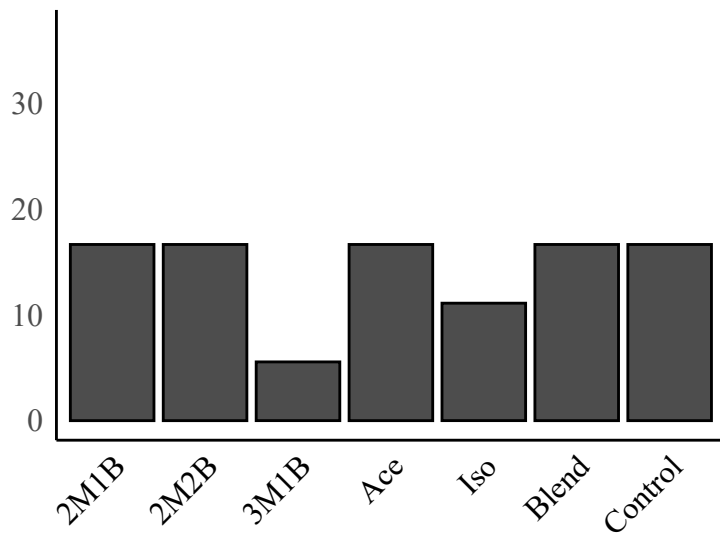

Supplement: Supplementary file 1 [file insects-16-00057-s001.zip › supplementary_materials/S1.pdf]

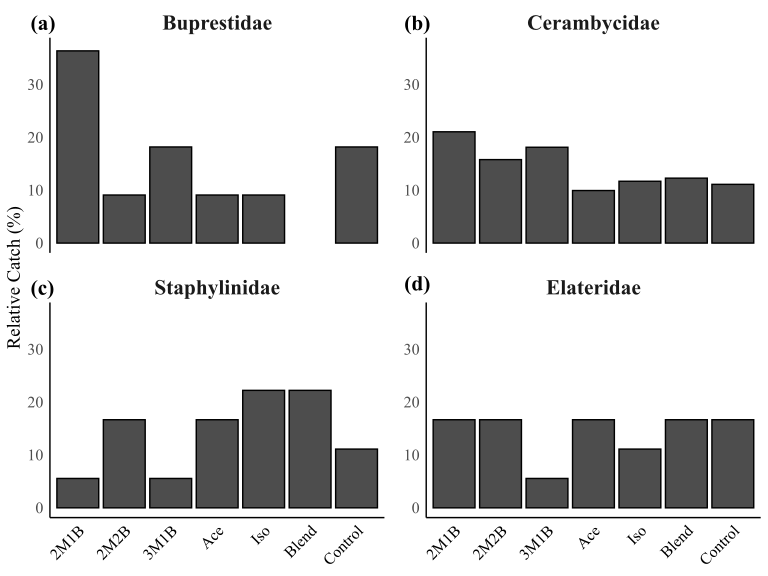

Supplement: Supplementary file 1 [file insects-16-00057-s001.zip › supplementary_materials/S1.png]

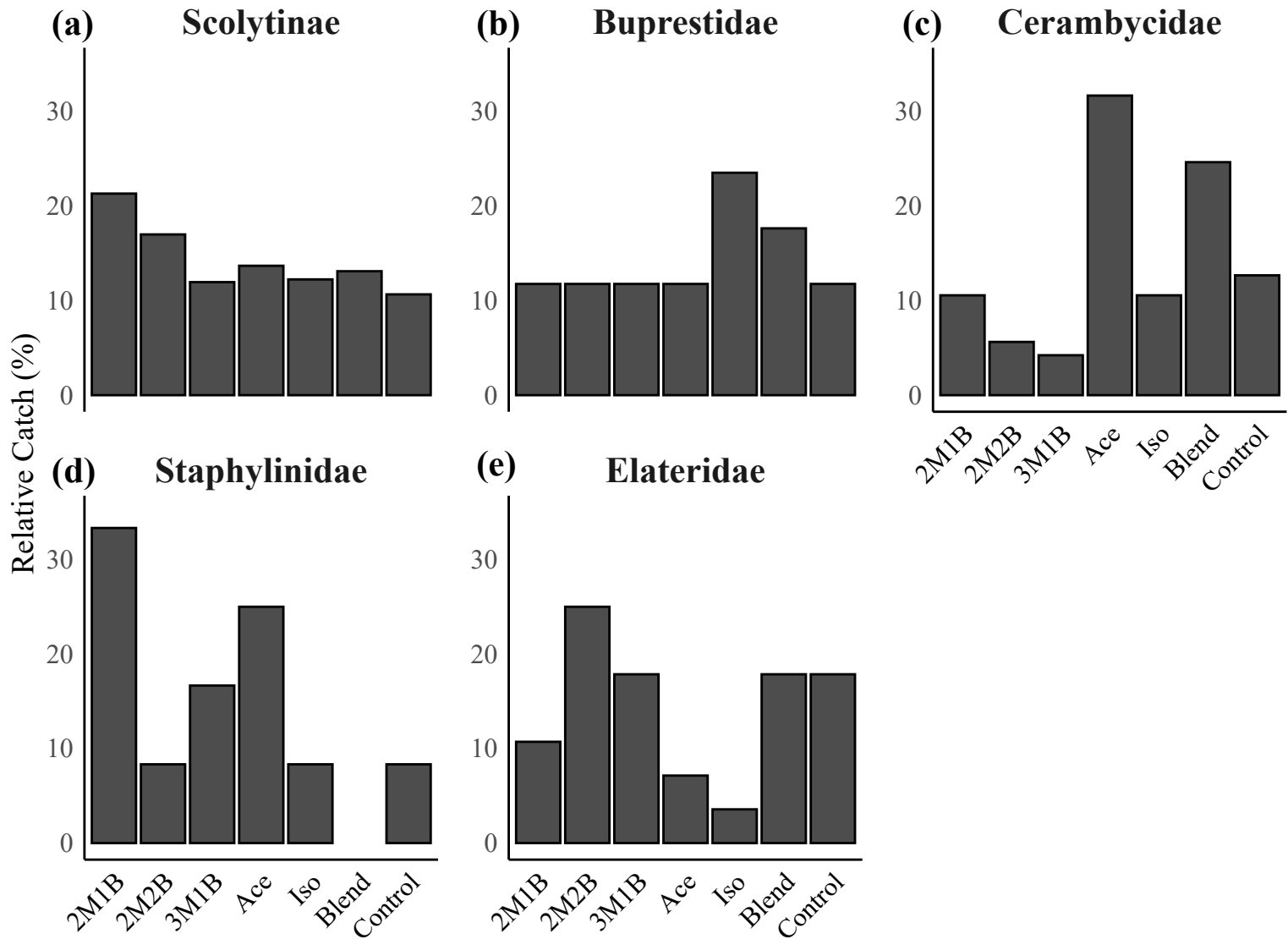

Supplement: Supplementary file 1 [file insects-16-00057-s001.zip › supplementary_materials/S2.pdf]

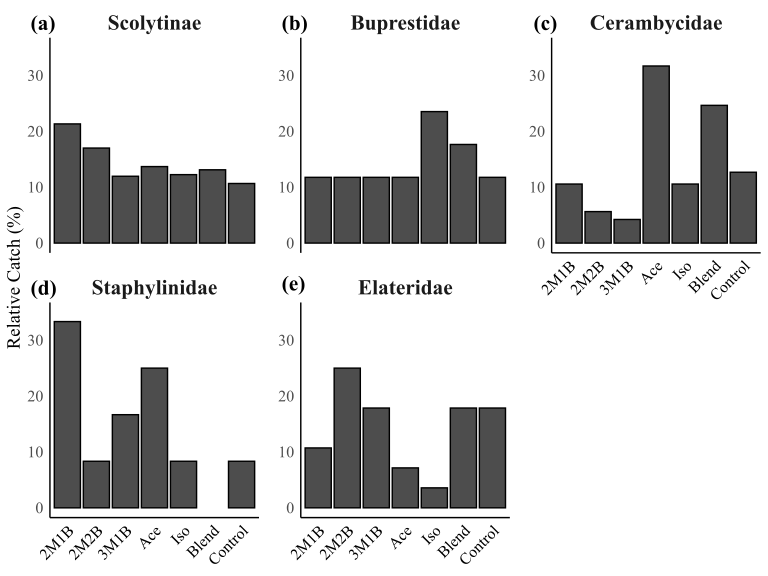

Supplement: Supplementary file 1 [file insects-16-00057-s001.zip › supplementary_materials/S2.png]

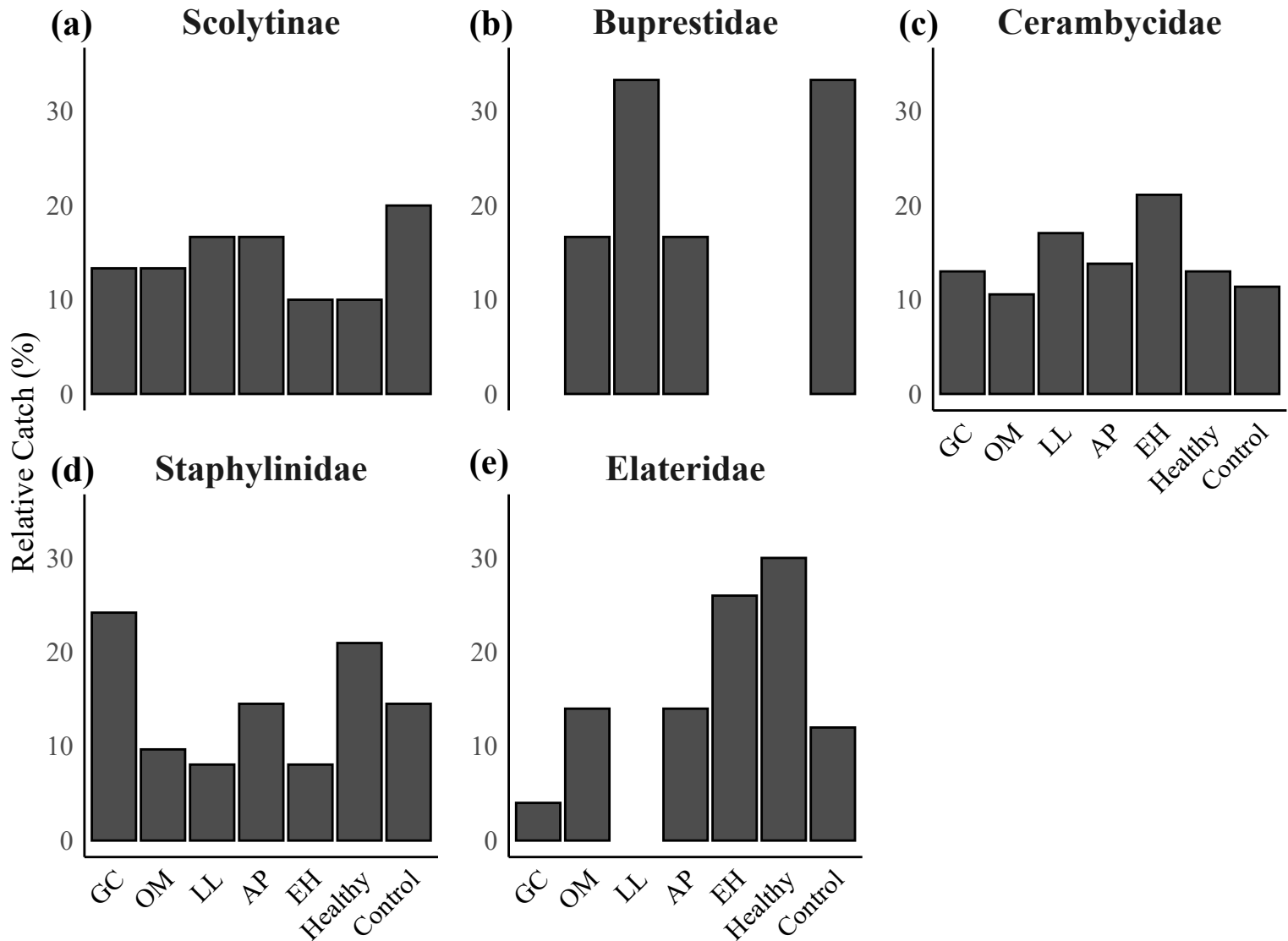

Supplement: Supplementary file 1 [file insects-16-00057-s001.zip › supplementary_materials/S3.pdf]

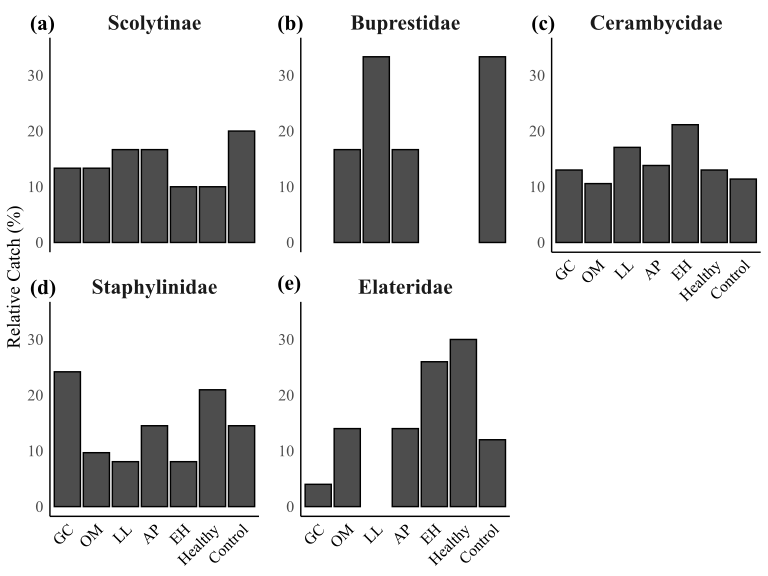

Supplement: Supplementary file 1 [file insects-16-00057-s001.zip › supplementary_materials/S3.png]
